# A Case of Letting the Cat out of The Bag—Why Trap-Neuter-Return Is Not an Ethical Solution for Stray Cat (*Felis catus*) Management

**DOI:** 10.3390/ani9040171

**Published:** 2019-04-16

**Authors:** Heather M. Crawford, Michael C. Calver, Patricia A. Fleming

**Affiliations:** Environmental and Conservation Sciences, College of Science, Health, Engineering, and Education, Murdoch University, Perth, WA 6150, Australia; crawfh01@gmail.com (H.M.C.); m.calver@murdoch.edu.au (M.C.C.)

**Keywords:** adopt, Australia, body condition, castrate, colony, diet, euthanasia, feral, predation, prey, shelter, spay, stray, Trap-Neuter-Return, TNR, wildlife, urban, vasectomy, welfare

## Abstract

**Simple Summary:**

Trap-Neuter-Return (TNR) is advocated as an effective, humane and ethical solution to problems caused by stray cats living in close association with human habitations. In Australia, TNR has previously been rejected by the Federal Government and the Australian Veterinary Association as an inappropriate management strategy for stray cats. Despite this, and public support for the control of cat numbers and legislative initiatives, calls persist for widespread trials of TNR. We review TNR literature that report empirical data to assess whether TNR resolves problems caused by stray cats and whether cats released under TNR would have a good quality of life. We identify ten ethical and welfare challenges that any cat control program must consider, particularly if cats are to be returned to urban environments. Simply, the weight of these data indicate that TNR cat management is unlikely to solve the problems in most cases and is unethical on animal welfare grounds. We argue instead for a holistic approach to reducing cat numbers using targeted adoption, early-age desexing and community education initiatives.

**Abstract:**

Trap-Neuter-Return (TNR) programs, in which stray cats are captured, neutered and returned to the environment are advocated as a humane, ethical alternative to euthanasia. We review the TNR literature in light of current debate over whether or not there should be further TNR trials in Australia. We revisit the problems arising from stray cats living in association with human habitation and estimate how many stray cats would have to be processed through a scientifically-guided TNR program to avoid high euthanasia rates. We also identify 10 ethical and welfare challenges that have to be addressed: we consider the quality of life for stray cats, where they would live, whether the TNR process itself is stressful, whether TNR cats are vulnerable to injury, parasites and disease, can be medically treated, stray cats’ body condition and diet, and their impacts on people, pet cats, and urban wildlife, especially endemic fauna. We conclude that TNR is unsuitable for Australia in almost all situations because it is unlikely to resolve problems caused by stray cats or meet ethical and welfare challenges. Targeted adoption, early-age desexing, community education initiatives and responsible pet ownership have greater promise to minimize euthanasia, reduce numbers rapidly, and address the identified issues.

## 1. Introduction

Cats (*Felis catus*) are flexible in their associations with people, and today they are classified and managed as pets, stray cats, or feral cats based on these interactions (see Glossary for definitions). Pet cats exist (or recently existed) on every continent, including some Antarctic bases, and stray and feral populations are established on every continent except Antarctica [1,2]. While the companionship of pet cats can have positive effects on human health [3], the ease with which cats transition to stray and feral populations [4] has, in many urban areas, exacerbated problems of depredation of wildlife [5,6]. Stray cats are also vectors of pathogens [7], with the potential transmission of rabies [8,9], *Toxoplasma gondii* [10,11,12] and *Sarcocystis neurona* [13] having been extensively studied. There is significant potential for the transmission of other pathogens [5,14], for example, the transmission of *Bartonella* bacteria to wild felids (e.g., puma, *Puma concolor* [15]). Stray cats are therefore a disease risk to people and domestic animals (including pet cats [7,16,17]).

Impetus for controlling cat populations comes primarily from recognizing that people are responsible for the support and survival of cats at high densities in urban areas. Densities of stray cats can be extremely high around areas where cats are deliberately fed or scavenging (where cats form ‘colonies’ [16,18,19]); for example, there are up to 2300 adult cats/km^2^ in Jerusalem, Israel [20]. At high densities, there is competition for food resources, space, and mating opportunities [1], and there are numerous public complaints about cat nuisance behavior (e.g., mating calls, fighting, urine-spraying, etc. [21,22,23]). Population control of stray cats therefore becomes necessary. Controlling stray cat numbers is also important for conservation of native species, as stray cats hunt, maim or kill substantial numbers of wildlife (e.g., [24,25]) and, in some locales, hybridize with wild cats (*Felis silvestris* [26]). As with free-roaming pet cats, stray cats in urban areas are at risk from road trauma [27,28], pathogens [29], deliberate or accidental poisoning [30], predation [31], and human persecution [32] while fending for themselves and not receiving veterinary care. For all these reasons, reducing the numbers of stray cats is an important goal for authorities and residents.

Options for controlling stray cats include chemical contraception, trapping and relocating cats to other locations, trapping and placing cats in animal shelters for adoption, trapping and euthanasia or Trap-Neuter-Return (returning cats to their original location; hereafter ‘TNR’ [33,34]) (see Glossary for alternative terms). At present, the main options in Australia are to trap and place strays in shelters, or trap and euthanize strays if they are ill and untreatable or if their temperaments are unsuitable for rehoming (e.g., [35]). Despite excellent rehoming rates in Australia, stray cats that enter shelters as sexually-intact adults are at higher risk of being returned to shelters and euthanized [36,37]. There is also evidence from Australia and overseas that cats who are not adopted quickly may eventually be euthanized from stress-related illness (e.g., upper respiratory tract infection [38]), if housing space, care, and funding are limited [39], or if they are fearful and/or aggressive [40]. High rates of euthanasia are a potential welfare issue for stray cats and for the professionals involved so, increasingly, some animal shelters and animal rights organizations advocate Trap-Neuter-Return to limit stray cat numbers, to theoretically improve their welfare by preventing euthanasia, reduce the resource burden on shelters/control groups and the emotional burden of euthanasia for people involved. Under TNR, stray cats are trapped, possibly medicated/vaccinated, and surgically neutered in a single trip to a veterinarian, before being released (usually) back at their initial point of capture. TNR typically focuses on colony cats that are deliberately fed and may be monitored by one or more caretakers (see Glossary). TNR is embraced as an *ad hoc* management strategy for local population control of stray cats in the United States of America (USA), Canada, Denmark, South Africa and the United Kingdom (UK) [33]. There are now advocates for TNR in Australia (e.g., [41,42,43]) despite the lack of endorsement by government at State and Federal levels and the Australian Veterinary Association [44,45].

Proponents of TNR are motivated by benevolence and a desire to save the lives of cats, and there is a perception that TNR could be the panacea for ‘solving’ issues of shelter overload (e.g., [42,46]). TNR proponents also claim that neutering will improve cat body condition [47], prevent intraspecific fighting and disease (such as Feline Immunodeficiency Virus; e.g., [48]), and that regular provision of food will reduce roaming [49]. TNR proponents also claim that returning neutered cats to their home range will reduce shelter intake and euthanasia [46,50,51] and ‘stabilize’ local populations by preventing the ‘vacuum effect’, whereby other cats enter the space vacated by euthanized cats (e.g., [52]). It is common for TNR proponents to make statements such as: *“When properly conducted, targeting control of a whole colony, TNR programs have proved to be effective in managing cat populations over many years and in many locations worldwide”* (p. 813 [53]) and that TNR is *“the only proven method to humanely and effectively control the free-roaming cat population”* (p. 37 [54]). Such claims are contested in the scientific literature (e.g., [5,55,56,57]), so the success of TNR is far from established.

There are calls to legalize TNR for controlling stray cat populations in Australia [41,42,43,58]; However, it is pertinent to ask if TNR addresses problems of wildlife conservation, public health and nuisance caused by stray cats, as well as delivering the population control and welfare benefits that proponents claim, or if it is ever likely to do so in Australia. In this paper, we review the TNR literature in light of current debate over whether or not TNR should be introduced in Australia for controlling stray cats living in urban areas (not ‘feral’ cats living in rural and remote regions of the continent; see Glossary). We estimate the numbers of stray cats that would have to be processed through a scientifically-guided TNR program to avoid high rates of euthanasia and then identify ten ethical and welfare challenges to be addressed by TNR managers overseas and by proponents in Australia. We consider the quality of life for stray cats, their impacts on people, pet cats, and urban wildlife. Finally, we consider topics specific to Australia, concluding that TNR cat management is unsuitable for Australia because it is unlikely to address the ethical issues that it sets out to solve, or the threats to wildlife, public health, nuisance and cat welfare arising from uncontrolled populations of stray cats. Targeted adoption, in which the effort and finances directed to the maintenance of TNR colonies is redirected to efforts to rehome stray cats, has greater promise to minimize euthanasia while humanely reducing the numbers of stray cats rapidly.

## 2. Population Control Under TNR Programs—Fundamental Issues

### 2.1. Do TNR Programs Successfully Deliver on Reduction in Stray Cat Numbers?

Research into TNR for cat management has increased dramatically since the 1990s [33]. Numerous ancillary studies contributing important knowledge about TNR programs have been published―such as gauging the motivations of caretakers [42,59,60], reporting public attitudes towards TNR and acceptability as a management strategy for stray cats [61,62], investigating cat health [63,64,65], the possible relationship between TNR and shelter intakes [46,51], developing population models [66,67], and determining the home ranges [68] and activities of cats in colonies [69,70,71]. Notably, however, there is still a dearth of robust evidence demonstrating the long-term success of TNR programs in reducing stray cat population numbers, both in the USA and overseas [44,72]. Modelling indicates that to reduce stray cat populations, TNR programs need to consistently neuter ≥75% of the fertile population for several years, which can be difficult to achieve [73].

**Figure 1:**
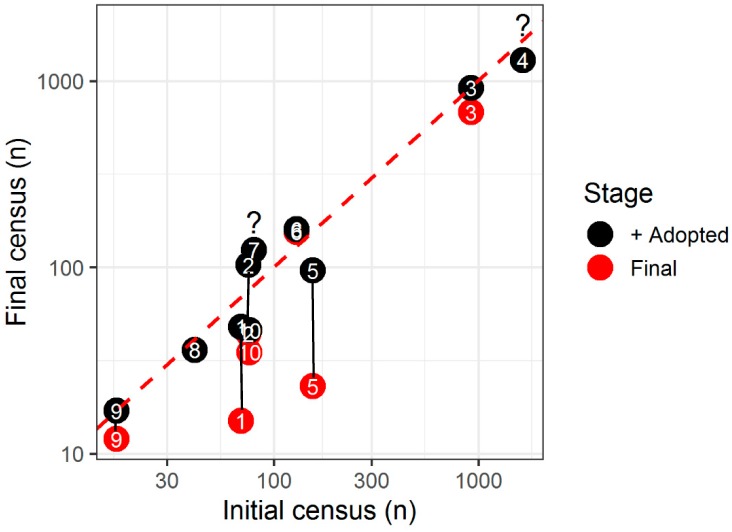
Comparison of TNR populations at initial census and final census for 10 of 11 studies (inadequate data for [79]). The red dashed line represents equality (i.e., no change in cat population over time). Values below this line indicate a decrease in population over time and data above the line indicate an increase in population over time. The vertical lines connect the final census data (red points) with the data for the final census plus numbers of cats that were adopted from the TNR program under study (black points). ? indicates that adoption data were not available for studies 4 and 7. Reference numbers as per Table 1.

The success of TNR programs is therefore contingent on demonstrating colony extinction or decreases in stray cat numbers over time. Only 11 published TNR studies (representing USA, Canada, UK, Israel, Italy and Australia) present data from an initial census (i.e., immediately before or at the time the TNR program commenced) and then a follow-up census (Table 1). Most did not manage to neuter all cats, and most euthanized a proportion of cats. The majority of studies were shorter than 3 years (range 1–11 years) and did not report extinction of colonies. Initial colony sizes (range 1–1655 cats, pooled across colonies), and final colony sizes (range 12–1293) are also hugely variable. Changes in cat numbers range from a 78% decrease to 55% increase (Table 1, Figure 1). The claimed ‘success’ of some studies in reducing cat numbers has been interpreted as ample evidence that TNR programs are effective. However, the number of cats that were adopted in these studies contributed markedly to the overall decrease and thus apparent ‘success’ of TNR (Figure 1 [72]). Proponents of TNR are increasingly acknowledging that high rates of adoption are required to reduce colony sizes and that extinction is unlikely (e.g., [74]). However, removing cats for adoption creates the very ‘vacuum effect’ that TNR colonies are supposed to prevent, with regular removals placing colonies in a permanent state of flux. There is therefore a fundamental conflict in the scientific principles of TNR in theory vs. reality.

Furthermore, all of the 11 TNR studies reported new cats joining colonies, some of whom were abandoned by owners (Table 1, four studies report immigration but do not provide raw data). These studies therefore demonstrate that colonies are rarely closed-populations and that the definition of a ‘stable colony’ is loosely interpreted by TNR managers (e.g., studies cited as stable by [75]). True stabilization is also contingent on continuous neutering of immigrant cats and requires regular finances. It is not possible to compare the changes in population size with efforts as only a few of these studies reported some of the financial and labor costs of their TNR program [64,76,77]. Reviewing these studies, management priorities are often unstated and lack target figures, and there is ambiguity in how to calculate population changes over time. The importance of such baseline TNR studies cannot be over-emphasized, particularly as a robust experimental design will enable program coordinators to budget time and labor and identify potential issues for long-term management actions. We also concur with Winter [78] in calling for accurate record keeping across TNR studies. Controlled experimental designs (e.g., [79]) are potentially the most powerful tool to assess the effectiveness of TNR, but they are scarce.

### 2.2. How Many Cats Would Be Saved from Euthanasia?

Annual euthanasia rates for stray cats processed by animal shelters can be extremely high. For example, in Ohio, USA, a survey of 165 control agencies indicated that 69% of 134,082 cats processed through their facilities in 2004 were euthanized [84]. In Australia, the RSPCA euthanized 27% of cats admitted nationally between 2016–2017 (*n* = 14,563/53,912); the causes of euthanasia were largely due to infections (24% of *n* = 14,563), other medical (23%) or behavioral reasons (25% [85]). In a recent survey of TNR colony caretaker respondents in Australia, Tan et al. [42] identified a median initial colony size of 11.5 cats (range 3 to >50, *n* = 44). If only cats with behavioral/temperament issues were saved from euthanasia, then 311 colonies (median size 11.5 used for our calculations) would be required annually, or up to 1266 new colonies if there were sufficient funds available to treat infectious and medical cases. However, shelter data can only provide tentative estimates of the wider stray cat population in urban areas. Legge et al. [86] estimated that, on average, Australian urban areas support 710,518 stray cats (‘unowned cats’, seasonal range 0.07–2.56 million) which would require 61,000 new colonies to accommodate these animals.

Barrows [56] claimed that indoor cats outlive free-roaming or feral cats 4–6 times. Considering a mean age of stray cats of two years [5], this would quickly amount to high densities of cats being returned to urban streets in Australia to be maintained for years. In the USA, Nutter [87] estimated that it would take 12.8 years for neutered cats in colonies to become extinct, and Levy et al. [75] reported a population of 155 TNR colony cats reducing to 23 over 11 years (i.e., reduction of 75%, 12 cats per year). Extinction of even modest-sized cat colonies is not quick and, while fewer cats may immediately enter shelters [50,51], labor and resources to maintain colonies for many years are needed. During this time, problems such as predation and public health risk persist.

These issues are typified in a recent Australian publication on TNR for managing strays living on campus at the University of New South Wales [43]. Funds from the university (AUD$9000) and partner charity (undisclosed value) were used to process 122 cats over 9 years. Feeding, trapping medicating and monitoring cats (including with motion-activated cameras), saved fewer than 50% of cats on campus from euthanasia (*n* = 48). Most of these were removed by adoption (*n* = 33, 27%). However, 71 cats were either euthanized (for disease, aggression and debilitation), died (from vehicle collisions, disease, neutering surgery and other *“accidents”*) or *“simply disappeared”* (p.11) during the trial (respectively *n* = 21, 17%; *n* = 15 cats, 12%; *n* = 35, 29%). The authors claim the study is a successful example of TNR in Australia and advocate for more studies. However, the manner and number of deaths surely refutes claims about improved cat welfare in the colony. We also feel that the study is a special case. The campus had a low cat density (one cat per 3.2 hectares), a low ratio of cats to people (1:1.7), there was constant fundraising (initial funds were quickly depleted), and an internal committee and governance structures ensured consultative decisions that could be enforced for the whole university community. Some other TNR studies have similar contexts (Table 1, Figure 1) and results, but they do not represent the majority of TNR programs operating or being introduced (see Challenge 1 below). We believe that sustained trapping and adopting friendly cats, ‘rehabilitating’ unsocialized cats (see Challenge 2) or euthanizing ill/aggressive cats would achieve similar results, without any of the public health concerns that can arise even within large, single employer environments if cats remain in the environment ([12], examples of university campuses in [5]). Removing cats instead of returning them also takes responsibility for the fate of the large proportion of cats who disappear after return to colonies.

### 2.3. How Much Would TNR Programs Cost?

TNR is expensive. Veterinary care and feeding cats before and after their release requires significant financial investment and time, for example (with no adjustment for inflation):

- Hughes and Slater [64] estimated that neutering 158 colony cats at a university veterinary teaching hospital in 1998 cost approximately US$9800 (discounted to US$4900). Blood tests, vaccines and sub-dermal microchips were donated (including surgical labor at 35 hours/student; number of students unspecified). The labor to trap cats was also donated (25–35 hours/person/week; trapping regime: 4–6 nights with 20 traps set/night every 4–6 weeks over two years). Traps were bought outright (cost US$900) and more traps were borrowed to meet the trapping effort required. Food was donated, as was the time for feeding/monitoring (approximately 15 h/person/week; number of people unspecified).
- Nutter et al. [88] estimated that the mean number of trap-nights required to trap 90% of adult cats (or until only one cat remained untrapped) in nine colonies was 8.9 ± S.D. 3.9 trap nights/cat.
- Webb [89] estimated that implementing TNR programs for approximately 150,000 stray cats across Melbourne, Australia, would initially cost AU$3 million to trap and neuter ($20/cat) and AU$39 million per year to feed ($5/cat/week).
- Lohr et al. [90] modelled the costs and benefits of TNR vs. trap-euthanasia in Hawaii and found that TNR was approximately twice as expensive to implement. Even TNR programs that used volunteers were more expensive than trap-euthanasia programs that paid professionals.

There is clearly substantial variation in the cost and labor required to implement and maintain TNR programs depending on the number of cats, intensity of trapping regime, volunteer support and provision of veterinary subsidies. Whether cats receive prophylactic treatment for parasites, etc., will also contribute to annual costs (see Challenge 5). Many programs rely on donated services, but good-will is not infinite, and authorities should not believe that TNR is sustainable without external investment in the efforts of private citizens to maintain cat colonies over years [42,43]. For charities, irregularity in public donations could jeopardize efforts to continue care over time.

### 2.4. Who Would Be Responsible for TNR Cats?

A major consideration in the welfare and ethics of maintaining cat colonies is who will be responsible for them, and what happens if that duty of care fails. Most cat colonies are maintained by private citizens who enjoy interacting with strays and feel responsible for improving the cats’ quality of life [42,91,92]. There may be no contingency plans for care of colonies should caretakers become financially strained, sick, die or move away. The Cat Protection Society (Victoria, Australia) identified the main issue with a trial of TNR cat management as long-term failure by caretakers who had assumed responsibility for ongoing colony maintenance [89].

Modest, localized surveys estimate that 10–26.5% of Australians provided/provide some aspect of care for cats that they do not own [91,93,94]. However, many of these caretakers (‘semi-owners’) fed/cared for cats that they knew to be owned by other people (59% and 44% of respondents respectively [93,94]). Caretaking of unowned stray cats may therefore be overestimated in Australian locales. The level and duration of care also varied between caretakers and location. For example, neutering of stray cats varied from 59% in Queensland to 16% in South Australia and 20% in Victoria (total *n* = 27/46, *n* = 5/32, *n* = 18/91, respectively [91,93,94]). Only 66% of caretakers fed cats daily, 66% did not intend to take full ownership of the cat, and 66% had provided care for <1 year [94]. Such variation in care by caretakers could undermine the implementation of TNR as a long-term strategy for improving cat welfare.

Caretakers may feed many stray cats and commit substantial time and finances [42,92,95,96]. Importantly, some surveyed caretakers felt they could not afford to feed a stray cat (16% [91]). Furthermore, additional cats (emigrants, abandoned cats, litters of kittens from cats that avoid trapping) can increase this load until personal resources are stretched beyond reasonable limits.

## 3. Can TNR Programs Improve Stray Cat Welfare in Australia?

Although there is substantial short-term appeal of TNR programs in terms of a proactive reduction in euthanasia, community values increasingly require consideration of the long-term welfare of stray cats as well as the ethical, social, public health and environmental impacts of implementing TNR for cat management. Here, we have identified 10 ethical and welfare challenges that would need to be considered in the context of TNR as a method for stray cat management, both overseas and in Australia:

1. Where would cats under TNR management live?
2. Is the TNR process itself stressful?
3. Would TNR cats be more vulnerable to injury?
4. Are stray cats vulnerable to high parasite loads and diseases?
5. Can parasites and diseases be treated in TNR cats? And at what cost?
6. Are TNR cats in poor health and body condition?
7. What would TNR cats eat?
8. Would TNR cat management impact people in urban areas?
9. Would TNR cat management impact pet cats?
10. Would TNR cat management impact urban wildlife?

### 3.1. Challenge 1. Where Would Cats Under TNR Management Live?

In the USA and UK, cat colonies are most commonly located in private backyards of citizens [18,59]. This is the same in Australia, where the most common locations for colonies are private or government homes/housing complexes (37% of 98 locations [42]). Clearly, the vast number of cats (smallest estimate is 1266 new colonies annually; see Section 2.2) cannot be maintained solely on private properties, especially as there is overlap between rescuing stray/abused animals and development of animal hoarding psychologies [97,98].

Under many TNR programs, cats are simply released back to their original point of capture. Tan et al. [42] reported colonies of stray cats around community areas such as industrial areas/factory complexes (20%) alleyways or streets (13%), schools and universities (7%), vacant blocks/buildings (7%), parks and reserves (5%), shopping centers (4%) and other locations (6%). However, maintaining colonies in such public locations rapidly becomes a community health and safety issue (reviewed by [14,99]) as well as a welfare issue for the cats themselves. TNR cats can have large home ranges: radio-trackers placed on sexually intact and neutered cats in TNR colonies on Catalina Island, USA, revealed that cats had average home-ranges of 1.5–2.1 km^2^ (150–210 hectares [68]). If cats under TNR management can roam freely, then they would move over whole neighborhoods, impacting the welfare of pet cats, wildlife and people, and may compromise stray cat quality of life. TNR programs that relocate cats from one location or colony to another, may subject those cats to aggression from other cats, competitive exclusion from the colony and/or food, and force them to investigate unfamiliar territory. The translocated cats may also introduce or contract novel pathogens.

Another option is to contain strays within ‘escape-proof’ fenced enclosures that keep cats safe whilst providing them with access to the outdoors and shelter, food and social interaction. These fenced ‘sanctuaries’ do exist [78], but have been rejected by some authors/charities as too costly for managing stray cats [33] or on welfare grounds [53], although we can find no scientific evaluation of sanctuaries. It seems contradictory to support the release of stray cats onto urban streets, where they cannot be guaranteed safety, access to food, veterinary care and humane euthanasia where required, over sanctuaries.

The decisions that TNR programs make about where to introduce or maintain cat colonies has the potential to adversely affect whole communities. Therefore, no TNR program should be attempted without environmental impact assessments, use of decision analysis networks [72], and consultation with the public based on factual information about possible consequences and expense. Where TNR is acceptable, the public should be kept apprised of efforts over time; however, program managers and volunteers must respect community decisions to reject TNR [100].

### 3.2. Challenge 2. Is the TNR Process Itself Stressful?

Capturing, transporting, neutering, vaccinating, worming and medicating are stressful procedures even for well-socialized pet cats [101], let alone for stray cats unsocialized/partially socialized to human contact. Trapped cats are transported to surgeries for processing [102] and often housed near other cats and/or dogs, etc. Cats trapped using bait should be held for 6–12 h before anesthesia to allow for digestion. Post-treatment, cats experience short-term and longer-term physiological and immunological suppression [103], are confused and distressed and stray cats, unlike pet cats, may not welcome the attentions of human attendants. High-turnover clinics may not have the facilities to house and monitor TNR cats for 24–48 h following surgery and may release cats back onto the streets without confidence in post-surgical recovery. There are no known studies of TNR impacts on longer-term recovery (e.g., incidence of acute or chronic infections, whether cats remain close to feed locations/avoid the area where they were captured for any period, or disruption of social relationships leading to relocation away from colonies—all of which may affect mortality). Fatalities during TNR procedures are reported to be low (0.2–0.7% [65]); however, Jessup [5] queried whether in the USA the veterinary care provided in TNR programs matched that expected of veterinary practices, specifically considering sterile practice, anesthesia, analgesia, and post-operative care. The stress of the TNR process is the likely cause of ‘trap shyness’, whereby cats are harder to trap a second time for health checks or further treatment (e.g., [64]). Without habituation to cat-carriers and handling, the ability of TNR groups to deliver post-surgical care (e.g., antibiotics) and prophylactic worming or vaccine regimes to every cat in a colony is possible but not guaranteed. The stress of the TNR process could also affect the fate of relocated cats. Teixeira et al. [104] reviewed literature on animal translocation and reintroduction programs and found program failure was related to animal stress, which can be experienced at any part of program execution and can be cumulative.

In our preferred alternative of placing stray cats in shelters for adoption, trapped cats are housed and monitored in shelters/clinics before and after neutering with follow-up care available. During these periods, cat temperament can be assessed, and the viability of ‘rehabilitation’ procedures determined. For example, cats that experience extreme stress upon entering shelters may become less fearful of people with regular positive interactions (e.g., regular bouts of stroking and vocalizing to anxious shelter cats over 10 days increased contentment, mucosal immune defense and reduced the incidence of upper respiratory disease [105]). Consistent handling procedures and enriched housing can also improve likelihood of adoption (e.g., the same staff cleaning cages, etc. [106]). Shelters that rehabilitate cats by desensitizing them to the presence of conspecifics may also be able to house multiple compatible cats together, which can improve welfare for some cats [107]. Strays that initially present as aggressive may actually be suitable pets if given time to adapt and positive attention [41]. This is confirmed by some TNR programs reporting that high proportions of stray cats are adopted (Table 1), but how TNR programs assess temperament or rehabilitate unsocialized cats (if they do), is rarely reported. The permanence of adoption and eventual fate of these TNR cats is also unknown.

Studies on adoptability, retention and fate of stray cats entering shelters vs. cats from other admission sources, are equivocal. For example, analysis of 12 months of shelter data from Ontario, Canada [108], revealed cats admitted as strays were less likely to be euthanized than surrendered pet cats. In contrast, Arbe Montoya et al. [37] found that cats acquired as strays and then surrendered to shelters in Queensland, Australia, were at higher risk of euthanasia than cats adopted from shelters and then surrendered to shelters.

With redirection of TNR funds and labor, there is clearly potential for animal shelters and TNR caretakers to rehabilitate some fearful cats and reduce the number euthanized by placing them in permanent homes rather than back onto urban streets. Rehabilitating cats might also improve the use and acceptability of cat sanctuaries to TNR proponents (see Challenge 1).

### 3.3. Challenge 3. Would TNR Cats Be Vulnerable to Injury?

Data for roaming pet cats suggest similar potential risks faced by stray cats in urban landscapes which, unlike pets, are unlikely to receive veterinary care. Pet cats can consume common poisonous plants such as lilies [109,110], and plants treated with herbicides (e.g., glyphosate [111]). Loyd et al. [112] recorded cats eating refuse, compost, roadkill and other dead animals, which can all make cats ill. Cats eating dead rodents risk poisoning from rodenticide [113]. Cats also drink from swimming and paddling pools, storm drains and puddles in car parking lots [112]. They are regularly brought to emergency veterinary centers having directly consumed hydrocarbons (e.g., fuel, paint), petroleum distillate (e.g., spirits), and pesticides [30,111]. Stray cats may be similarly vulnerable to ingesting toxic compounds (particularly if they are young, hungry or ill), and as cats often hide when traumatized or ill [1,101], strays are less likely to attract ministration.

Trauma, especially that caused by vehicle collisions, is extremely common in free-roaming pet cats [114,115,116,117]. Mortality statistics for stray cats would likely be even greater because they are unlikely to receive immediate veterinary attention [28]. For example, 18% of 164 cats in TNR colonies were killed by vehicles [87]. Roaming cats may also be injured/killed by other carnivore species (e.g., dogs, coyotes, etc. [118,119]).

Neutered or not, cat colonies attract other cats (including cats abandoned by their owners) and more fighting occurs as densities increase [120,121]. For example, of the 1659 cats entering a Melbourne shelter with injuries, 79% originated from colonies [35]. The costs of treating injuries or removing nuisance individuals from colonies quickly rises for conscientious caretakers. Gunther et al. [21] found that 54.5% of calls about stray cats made to councils in five cities in Israel were complaints about decomposing cat carcasses; an additional 16.5% of callers reported injured cats (total calls to cities about stray cats *n* = 101,415).

Intentional cruelty towards cats varies with cultural attitudes and era (reviewed by [122]). Whatever the motivation (e.g., retaliation, boredom, psychopathology), reports to welfare organizations are common. In just over two years, 65 cats (2% of *n* = 3156) presented to a diagnostic imaging center in Croatia with metal projectiles in their bodies (e.g., air-gun and shotgun ammunition, as well as a homemade arrow [32]). Almost half of these cats (40%) had new or old associated fractures. High densities of stray cats may attract negative public attention (i.e., reporting nuisance behavior [18,21,22]), decrease social tolerance towards cats, and increase incidence of cruelty.

### 3.4. Challenge 4. Are Stray Cats Vulnerable to High Parasite Loads and Diseases?

According to Pedersen [123], each cat population has its own viral, bacterial, parasitic and protozoal flora. Cats within a given area are most resistant to the pathogens to which they are continuously exposed, and this is especially relevant for cats living at high densities. Sharing of fomites (e.g., food bowls [96]) and interchange between cat populations is also likely to spread pathogens to other cats/populations. Colonies fed by caretakers encourage the presence of cats (including pets), facilitating the spread of parasites and diseases. Although infectious diseases are more serious in juvenile cats, adult cats carry the diseases affecting younger animals. For certain parasites and diseases, infected adult cats are less likely than juveniles to exhibit a decrease in physical condition. Therefore, removing juvenile cats and returning wormed/vaccinated adult cats to colony locations does not necessarily reduce or prevent reinfection by pathogens.

Variation in the health of stray cats is expected for different locations and climates. For example, prevalence of endoparasites can range dramatically amongst stray cats (Table 2). A study of 113 stray cats in Iran identified 15 species of endoparasites, with 97% of these cats infected [124]. Similar examples of high prevalence exist for stray cats globally (e.g., 91% of stray cats in Zealand, Denmark [125], 83% for Doha, Qatar [126], 91% for Lisbon, Portugal [127]). Such high prevalence compromises health.

The methods used to quantify stray cat health are also likely to vary in diagnostic accuracy. An observational study of 210 colony and 253 stray cats in Auckland, New Zealand [128], found that more than 80% of cats displayed no signs of ear crusting or nasal and ocular discharge and more than 90% had no obvious signs of injury. In contrast, laboratory testing and physical examination of cats congregating at rubbish dumps at four locations in the Australian Capital Territory and State of New South Wales, Australia, found that many suffered from viral disease and multiple bacterial infections [129]. Nearly 80% of cats tested had Feline Immunodeficiency Virus (FIV; 79% of *n* = 83 cats tested), and there was a high prevalence of gingivitis (64%), throat conditions (54%), and eye conditions (23%) which are indicative of cat flu (Feline Herpesvirus or Feline Calicivirus). Studies of parasites and diseases clearly show that stray cats are vectors for many pathogens and this merits location-specific assessment of stray cat health using multiple assessment methods where possible.

#### 3.4.1. Ectoparasites

Cat fleas, ticks, mites and lice are common ectoparasites of stray cats [145,146,147,148]. Ectoparasite species and prevalence varies with geographic location, season and the suite of animals roaming cats encounter (e.g., dogs, raccoons, etc. [149]). Ectoparasites cause skin disease and allergies, move between individuals and species, and infect hosts with blood-borne parasites (e.g., fleas introduce zoonotic *Rickettsia felis*, *Bartonella henselae* and *Toxoplasma gondii* [150,151,152]). These pathogens cause life-threatening anemia in young or immune-compromised animals [149]. Cats often host communities of ectoparasites (e.g., [153]), although this is not always reflected in their body/coat condition.

#### 3.4.2. Gastrointestinal Parasites

Gastrointestinal parasite burden can be high in stray cats (Table 2). For example, an average of 53 tapeworms (*Joyeuxiella echinorhyncoides*) and 17 roundworms (*Dipylidium caninum*) were recorded for stray cats from Iran [124], while an average of 286 roundworms (*Ollulanus tricuspis*; up to 2877 individuals) and 42 tapeworms (*D. caninum*; up to 297 individuals) were recorded for stray cats from Lisbon, Portugal [127]. Parasite burden varies with age, health and living environment (e.g., humid warm environments increase survival of parasite egg stages in fecal samples and soil). The feline tapeworm (*Taenia taeniaeformis*) has high prevalence in stray cat studies globally (Table 2). Cats are a definitive host for *T. taeniaeformis* and, as such, can tolerate a large parasite load without symptoms. For extremely high burdens, clinical signs can include malaise, capricious appetite, colic and mild diarrhea. Intussusception or blockage of the intestine, emaciation, and seizures may occur. The cat roundworm (*Toxocara cati*) is another common endoparasite (Table 2 [154]) that is ingested when cats consume rodents (paratenic hosts), and its larvae can be transmitted to kittens via the trans-mammary route. Another common tapeworm is *D. caninum*, transmitted by fleas, and promoted through high population densities and group living. Although common, these three endoparasites are difficult to diagnose in stray cats without thorough microscopic examination of feces. These parasites are also transmitted to other species, so the need for prophylactic control in stray populations should not be dismissed on the basis of physical appearance alone.

#### 3.4.3. Haemoparasites

Cat are definitive hosts for *Toxoplasma gondii*, a blood-borne parasitic protozoan which they contract through eating raw meat, rodents or birds. Many caretakers feed stray cats with raw meat and may inadvertently play a role in the transmission of *T. gondii* and other pathogens [155,156]. The parasite is difficult to detect because juvenile cats normally excrete oocytes for short periods, and it is rare for adult cats to excrete oocytes [155]. Through serum analyses, however, it is evident that stray cats show a high infection rate. The prevalence averages 30% [157], but 85% of 48 stray cats from Addis Ababa, Ethiopia, tested positive for *T. gondii* [158]. In Tehran, 90% of stray cats tested positive [159], while the infection rate was 31% for stray cats from Guangzhou, China [160]. Miró et al. [161] found that stray cats in Spain had a higher prevalence of *Toxoplasma* than pet cats (36% vs. 25.5%), adults had a higher prevalence than juveniles (37% vs. 14%) and males had higher prevalence than female cats (45% vs. 32%). *Toxoplasma* has also been detected in stray, pet and feral cat populations in Australia [162,163,164]. Although *Toxoplasma* is rarely fatal for cats, it does infect other species and health consequences are often unknown (e.g., [165]). Implementing TNR programs may facilitate proliferation of *Toxoplasma*.

#### 3.4.4. Viruses

Stray cats are vulnerable to several naturally occurring viruses, some of which are species-specific [166]. FIV is commonly reported in stray cats (reviewed by [48]), with up to 45% of cats harboring the virus [129]. FIV attacks the immune system and clinical signs include fever, anemia, lymphadenopathy and weight loss. FIV infection induces a long asymptomatic stage (lasting months to years) followed by an AIDS-like syndrome. Recovery is not possible, but not all infected cats develop the active form of the disease. FIV-positive cats can have a poorer general condition and are more prone to repeat eye and respiratory tract infections (e.g., conjunctivitis and cat flu, [129,167]). FIV is transmitted via saliva through bites [168]. Adult males are more likely to contract the virus when competing for territory, females and food [169,170], and bold temperaments also play a role in transmission [29].

Feline Leukemia Virus (FeLV) is associated with malignant lymphoma, anemia, liver degeneration and immunosuppression (reviewed by [171]). Most cats with FeLV recover, develop immunity, and do not become excreting carriers, whereas others become persistently viremic. After a variable asymptomatic stage, persistently viremic cats develop a FeLV-related disease and die [171]. The virus is shed in cat saliva, nasal secretions, urine, feces, and milk [171]. Kittens and cats living in groups or high densities (including colonies) are particularly susceptible, as any direct contact spreads the disease [168].

Feline Parvo Virus (FPV or Panleukopenia Virus) is spread by direct fecal–oral contact, and indirectly following contamination of the environment or fomites (e.g., food dishes, grooming brushes, etc.; see review by [172]). Diagnosis is possible from whole blood and fecal samples. FPV is rare but extremely contagious. Infected cats excrete the virus for at least six weeks post-infection; it survives in the environment for several years. Clinical symptoms include fever and hemorrhagic gastroenteritis, but viral replication is rapid, and cats may die before exhibiting symptoms. Mortality rates are >90% in kittens, and those infected in utero or in the first month of life can develop incurable cerebellar hypoplasia that retards development of finer motor skills.

Cats are the most commonly recorded animal with rabies in the USA, although the recorded incidence is low (4% of cases in non-human animals in 2015 [8]). Of the 23,101 cats tested for rabies in 2015, 244 (1%) were confirmed rabid; 40 of 42 (95%) animals with a vaccination status had no record of rabies vaccination, but one had expired status, and another was up to date. Nevertheless, Jessup and Stone [9] reported that human exposure to rabies is more commonly caused by cats than other domestic animals; between 1993 and 2002 in New York State, cats accounted for one-third of cases of human exposure to rabies. TNR simply returns potential rabies hosts to the environment.

### 3.5. Challenge 5. Can Parasites and Diseases Be Treated in TNR Cats? And at What Cost?

If TNR caretakers wish to improve stray cat health, the out-of-pocket expense could be high. For example, in Australia, annual treatment of ectoparasites and worms in stray cats using an all-in-one ‘spot-on’ liquid application would cost a minimum of approximately AU$157/cat (Table 3) and require monthly capture of each colony cat. There can be issues administering oral treatments as cats need to eat tablets in food and receive an effective drug dose (standardized by body mass); in Australia this would annually cost AU$150/cat (Table 3). In addition to repeated handling of the animals, a vaccination and worming program also requires continuous funding/donations.

Vaccines provide protection from the primary feline viruses (Table 3). Vaccination should be administered following thorough blood screening (e.g., giving FeLV vaccine to an animal that is already positive for the virus has no benefit [171]). However, the rapid processing of stray cats under TNR (neutering and vaccinating large numbers) is not always conducive for testing cats for diseases or internal parasite burden. For example, only three out of seven TNR programs surveyed in the USA tested for FeLV or FIV before releasing cats, and these tests were optional [65]. Other studies only tested cats for FIV and FeLV if they appeared to be sick, were mature males, or candidates for adoption [75,76] and, given that cats with these diseases do not always exhibit symptoms, the usefulness of testing some cats (or none) is questionable. Even if strays are identified as carrying disease, there may be little intention to euthanize them, with many strays re-released to further transmit the disease.

Furthermore, some virus protection requires regular vaccination boosters that necessitate follow-up trapping of cats, which may be difficult or impossible. Hughes and Slater [64] report that only 23 of 80 TNR cats (29%) due for follow-up vaccination were able to be re-trapped and vaccinated―20 within 3 months of vaccination due date, two were overdue by 6 months, and one was overdue by 8 months. Instead of removing strays from the streets permanently, TNR programs choose to focus resources on neutering and hope that there will be a corresponding decrease in fighting and thus a decrease in spread of FIV and FeLV [74]. Whether this decision is the most ethical for infected cats is debatable.

### 3.6. Challenge 6. Are TNR Cats in Poor Health and Body Condition?

Unhealthy stray cats may be excluded competitively from food sources, have shorter life-spans and, if reproductive, may have higher juvenile mortality. The health of stray cats entering shelters affects their fate. For example, colony cats admitted to a Melbourne shelter were typically thinner (46% were underweight or emaciated) and in poorer health than surrendered pets and other admissions (65% had cat flu, etc. [35]); and poor body condition was a predictor of euthanasia outcome for these animals. Although TNR proponents claim that colony cat body condition increases following neutering [47], tested sample sizes are small and measured fat deposits not nutritional or immune health. Given that TNR cats are hard to recapture following neutering (e.g., [64]), their health status is generally inferred from visual assessments (e.g., [96]). For example, Zito et al. [128] visually assessed and compared the physical condition of stray and colony cats (body condition, coat condition, nasal and ocular discharge, ear crusting and injuries) in New Zealand and concluded the majority of cats were in excellent health. However, the authors acknowledge that visual assessments alone do not allow one to make *“accurate inferences about… the true welfare status of these cat populations”* (p. 4). Alone, visual assessment of TNR cat body condition is unlikely to detect all health issues (e.g., parasites, bowel blockages, mastitis), especially if cats become cryptic when ill or do not display sickness behavior [173]. In some studies, poor physical conditions of TNR cats are easily recognizable [174]. For example, in San Paolo, Brazil, 21% of strays were underweight, blind, had skin problems or scars [175]. More than a quarter of stray cats living in a population of >250 in Botany, Australia, are blind, injured, or ill despite being fed regularly and provided with shelter [176]. The health and body condition of stray cats is therefore multifactorial and site-specific (see Challenge 4) and management programs should thoroughly and regularly assess stray cat health, using multiple methods. This will require regular finances and labor.

### 3.7. Challenge 7. What Would TNR Cats Eat?

Many proponents of TNR programs believe that the regular provision of cat food (commercially developed to optimize nutrition) will reduce scavenging of anthropogenic refuse by colony cats. However, refuse offers cats valuable opportunities to obtain food without expending energy on active hunting and may comprise a substantial portion of the diet of stray cats. For example, a study of 97 cat fecal samples on a Brazilian university campus identified 21% vegetable matter and 15% non-food items (i.e., refuse) in winter and 18% vegetable matter and 15% non-food items in summer [177]. In Israel, 43 stray cats sampled from urban settlements had eaten only human food or cat food (across the country stray cats are deliberately and regularly fed), and 69% of stomach volume from 59 stray cats in more rural settlements was *“trash”* (p. 130 [178]). Rees [18] surveyed caretakers of 339 colonies in the UK and found that although 92% of colonies fed cats daily, refuse was a source of food for 66% of cats. In Australia, a 2-year study of cats around a rubbish tip in Victoria [179] identified refuse in 80.5% of 159 fecal samples; 14 refuse items were identified, including plastic, foil, cloth and paper. Some refuse clearly offers cats little nutrition (e.g., plastic, aluminum foil, etc.) and may compromise their immediate health (e.g., bowel blockages or ruptures), or long-term health if consumed regularly/exclusively (e.g., [180]). Responsible care of any cat colony must ‘clean up’ the surrounding environment to minimize scavenging opportunities. This will require extra time commitment and labor from caretakers and possible collaboration with waste collection authorities.

### 3.8. Challenge 8. Would TNR Cat Management Impact People in Urban Areas?

#### 3.8.1. Zoonotic Parasites and Disease

Robertson (p.367 [34]) stated: *“There is concern about the possibility of cats transmitting diseases to humans, but in reviewing the literature there is little information on the actual frequency of zoonotic diseases in which cats can be implicated”*. However, given the growing literature showing that stray cats globally host a range of zoonotic parasites (Table 2), and limited data on the frequency of transmission, risk management is prudent [181].

TNR programs in populated areas effectively increase spatial proximity between cats and humans which may increase zoonotic transmissions of parasites [124]. For example, cats regularly defecate and urinate in parks, sand boxes and play areas used by children and adults [182,183]. In urban areas with stray cats in Prague, prevalence of roundworm eggs (*T. cati*) in soil reached 45% of all samples [184]. Roundworm larvae migrate through viscera and the eyes, causing human toxocariasis [157]. Hookworms (principally *Ancylostoma* spp.) are transmitted by skin contact with soil contaminated with larvae, which burrow into skin causing cutaneous larval migrans [157].

Toxoplasmosis infects humans worldwide, with nearly one-third of adults seropositive in the USA and Europe [157], mostly as a result of eating raw or undercooked meat [155]. While not all *Toxoplasma* is contracted directly from cats, handling cats and their waste (deliberately or accidentally) is directly linked to fetal abortion in humans [157].

Rabies is a familiar zoonotic disease spread by feral cats [157]. In 1995, >82% of 228 human victims given post-exposure prophylactic treatment administered in the USA for possible exposure to rabies resulted from contact with feral or stray cats [185]. TNR programs can be complicit in the transmission of zoonotic diseases. For example, Jessup and Stone ([9] p.495) reported that: *“The largest TNR program ever conducted in California resulted in the release of 90,000 feral* (stray by our terminology) *cats not vaccinated for rabies.”* They further noted that booster vaccinations and post-bite re-vaccinations were unachievable for stray cats. Rabies is currently not present in Australia, although this is believed to be only a matter of time [186].

Additionally, cats transmit plague to people [187] and laboratory studies show that cats exposed to avian flu (H5N1) contract the disease and shed the virus extensively, raising concerns about cats as vectors for a pandemic [188].

#### 3.8.2. Public Nuisance

**Figure 2:**
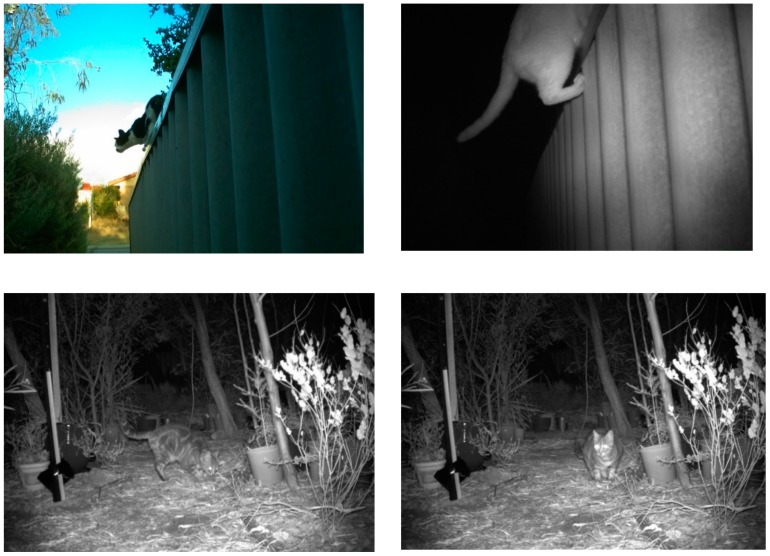
Evidence that free-roaming cats engage in nuisance activity on private properties. Top left and right: Two stray cats enter and exit the front yard of a private property by climbing over a boundary-fence. Bottom left and right: A stray cat defecates on a garden path in a suburban backyard. (in Western Australia, stray cats are any cat without a collar, etc. [191]). Photographs: Heather M. Crawford.

The nuisance activity of roaming cats is regularly reported to councils (e.g., [21,46]) and fuels neighborhood disputes [189]. The nuisance activities of cats are particularly difficult to curtail considering their typical nocturnal activity patterns and ability to evade physical barriers and gain access to private property (Figure 2). In trials of ultrasonic cat-deterrents in suburban backyards in Perth, Australia, Crawford et al. [190] detected 78 ‘unwelcome’ cats encroaching onto 17 private properties. These cats engaged in numerous nuisance activities, including defecating and urine scent-marking (Figure 2). Pairs of cats fought or played together, and 20 hunting events were recorded (at least four cats successfully caught prey). Approximately half (47%) of the nuisance cats appeared to be unowned (did not wear collars as is required by State legislation [191] or were not confirmed as belonging to a local resident), with at least 38% of unowned cats confirmed to be male. Even with novel deterrents, stray cats can exacerbate tensions between neighbors and caretakers, and this may increase complaints to local councils who may need to allocate funds to dispute resolution.

It has been argued that neutering may reduce home ranges and that TNR cats may therefore attract fewer complaints and encounter fewer dangers from roaming. However, it is actually very difficult to demonstrate whether or not neutering changes ranging activity because home ranges are naturally variable, being influenced by cat sex, age (and possibly also age at neutering), housing density, and social relationships between cats [68,192,193,194]. Neutering colony cats is also believed to reduce fighting and therefore prevent injury and disease. Although, Hart and Barrett [195] reported that 53% of male cats (*n* = 42) immediately decreased fighting after neutering, and 35% decreased gradually after neutering, no change was perceived in 12% of cats. In another study [196], fighting in males did not differ between cats neutered pre-pubertally or in adulthood, because fighting is influenced by social dynamics not just sexual status. Neutering stray cats therefore does not guarantee that roaming, fighting or public nuisance complaints will decrease [23].

#### 3.8.3. Physical Injury, Waste and Allergies

The tolerance of cats towards humans depends on exposure to human activity from an early age and the friendly nature of interactions [197]. Cat temperament is also influenced by that of the tom cat [197]. Wild, aggressive or fearful cats are least likely to be adopted, and therefore their alternatives are euthanasia or release (under a TNR program). For example, colony cats admitted to a Melbourne shelter were significantly more *“actively antisocial”* (p.199 [35]) than cats from other sources (e.g., pets), and low sociability was the main predictor for euthanasia outcome. Unsocialized cats are also difficult to trap, and difficulty increases with subsequent attempts to trap [68,87]. Their repeated capture for husbandry or veterinary treatment will become increasingly problematic for TNR caretakers [64].

Cats that are unsocialized or not tolerant of the presence of people can be unpredictable and dangerous. In Israel, 3354 complaints, were made to five city councils about aggressive cats over five years [21]. Barrows ([56] p.1367) documents numerous cases in the USA of rabies alerts, followed by shutdowns of public spaces and extensive human treatment. Colony caretakers are often older people, for example, the median age of feeders of free-roaming cats in Israel was 58 years [63]. Cat bites and scratches cause soft-tissue trauma that readily infects [198], and older people may be at greater risk of injury. For example, in 2016–2017, 130 people from South Australia were admitted to hospital with injuries from cats, with the majority older than 55 years of age [199].

Like free-roaming pet cats, TNR cats also pose a risk to the safety of motorists who may swerve or brake heavily to avoid a collision. Given the high prevalence of road accident trauma to cats in general (e.g., [27,28]) and colony cats in particular (e.g., [87]), the risk of injury or death to motorists, and cats, should not be ignored in assessment of the appropriateness of TNR.

Large or numerous cat colonies will introduce substantial urine and feces into the environment. Dabritz et al. [10] estimated that in three Californian communities, 2309 roaming cats contributed approximately 108 tons of feces to the environment annually. The effects of adding such quantities of waste into urban environments are unknown (i.e., water quality, nutrient flow). Additionally, cat colonies will introduce more cat dander and fur into the environment. Dander allergy and asthma are common reasons for surrendering pet cats to shelters [200,201]. Research is needed to determine whether humans living in proximity of cat colonies are at risk of adverse health effects.

### 3.9. Challenge 9. Would TNR Cat Management Impact Pet Cats?

**Figure 3:**
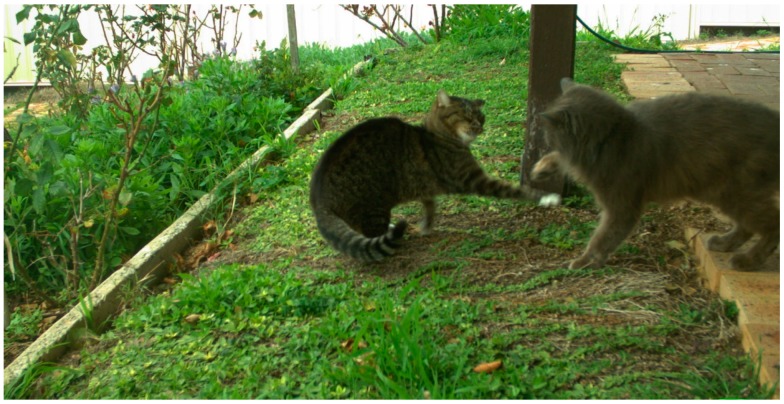
Evidence of cats conflicting with pet cats: a stray cat (any cat without a collar, etc., [191] in Western Australia) interacting with a pet cat in its owners’ backyard (pet cat on left). Photograph: Janine Kuehs.

Stray cats encroaching into private backyards can intimidate, steal food or fight pet cats in their home range [1] (Figure 3). Colonies attract other cats, including pets (e.g., [43]). In both situations, and regardless of neuter status, fighting and disease transmission can result. The mouths and claws of cats contain myriad pathogens that can compromise the health of fight-victims [202,203]. In Sydney, Australia, the prevalence of FIV in 48 colony cats and 20 TNR cats was 21% and 25% respectively [17]; higher than the 16% prevalence in 169 pet cats with access to the outdoors. In Perth, Western Australia, a study of 418 fecal samples from cats, pets had four times lower prevalence of gastrointestinal parasites compared with refuge cats and kittens (2% vs. 8% [204]). The degree of contact that cats had with other cats (and dogs) significantly influenced the prevalence of parasitic infection [204]. Therefore, even if pet cats are regularly vaccinated and dewormed, TNR may increase urban cat densities overall and increase the likelihood of cats fighting and being exposed to common or novel pathogens.

### 3.10. Challenge 10. Would TNR Cat Management Impact Urban Wildlife?

Cats have an extremely strong innate hunting instinct, so it cannot be assumed that because stray cats are fed, their impacts on local wildlife will be minimal [43,91]. Despite being fed once or twice daily, many pet cats hunt wildlife and take prey back to their core range or owner’s home [205]. Similarly, laboratory studies confirm cats will hunt rats in preference to eating their regular commercial foods [206]. Hernandez et al. [70,71] used video cameras attached to collars to quantify the activity and hunting of cats living at 11 regularly-provisioned TNR colonies in Georgia, USA. Unsurprisingly, cats spent most of their time resting or sleeping (89.5%) and spent 0.6% of time eating at food stations within the colony. However, 9% of time was spent exploring the environment and 0.9% of time was spent hunting [70]. While the percentages for exploring and hunting seem small, footage revealed that 24 of 29 collared cats hunted (i.e., stalking, harassing, capturing prey [71]). Across an average of only 22 hours of video footage per cat (range 3.8–60 h), 18 cats were recorded killing 174 animals (average 9.6 animals/cat, max. 65 killed by one cat). The average number of hunting events was 9.4/day with a 44% success rate that varied with prey taxa (82% success for orthoptera, 76% amphibian, 69% reptile, 64% mammal and 17% avian). Colony cats therefore clearly hunt despite regular provision of food, and their impact on wildlife populations could be substantial, including prey that are eaten or abandoned when dead or injured [207], and that sub-lethal effects are also likely to occur (i.e., if cat presence deters or negatively influences the behavior and breeding of species such as song birds [208]).

While the impact of stray cat predation on wildlife has received less attention than pet or feral cats, Jessup [5] documents studies from the USA that note reduced populations of native bird species, including complete absence of ground foraging species, near sites where unowned cats were fed. It is likely that further studies of stray cat predation will add to documented predation by pet cats in urban areas. For example, modelling has established that some urban areas of New Zealand are population sinks for some bird species because of cat predation [209]. In urban/peri-urban areas of Australia, documented examples of wildlife threatened by cat predation include the Vulnerable [210] striped legless lizard, *Delma impar*, in suburban Canberra [211]; and the Endangered [212] eastern-barred bandicoot, *Perameles gunnii*, in Hamilton, Victoria [213]. A further 19 mammal, 59 bird, 24 reptile and seven amphibian species have been recorded as prey of pet cats [214,215,216,217,218,219].

**Figure 4:**
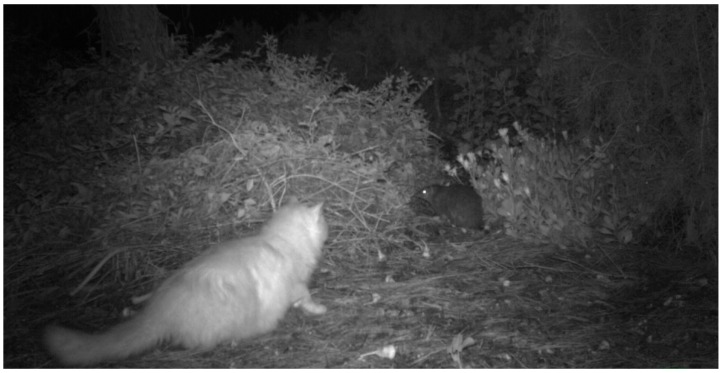
Evidence of cats conflicting with wildlife: a stray cat (in Western Australia, any cat without a collar, etc. [191]) stalking a quenda, an endemic species of bandicoot (*Isoodon fusciventer*) in an urban bushland reserve. Photograph: Janine Kuehs.

Australian cities contain more threatened animal species per unit area than non-urban areas [220]. For example, Bryant et al. [221] documented the persistence of native quenda (*Isoodon fusciventer*, an endemic species of bandicoot) living in Mandurah, Western Australia; however, roaming cats stalk and kill quenda ([190,218] Figure 4). Another study showed that a single, well-fed pet cat drove the local extirpation of a *Ctenotus* sp. lizard population [222]. Minimizing further anthropogenic impacts on wildlife populations persisting in urban areas includes introducing measures that reduce predation by stray and pet cats. Various predation deterrent strategies have been trialed (e.g., bells [223,224], pounce protectors [216,224], auditory deterrents [190,225,226], curfews [227,228], buffers [229]) with mixed success. In Australia, the impact that even small TNR colonies could have on endemic or range-restricted fauna therefore cannot be discounted. Trials of TNR to determine predation impact on these species should not be risked.

Control of pest populations could be argued as motivation for maintaining cat colonies (e.g., [54]). Rats (e.g., black/ship rat, *Rattus rattus*, brown/sewer rat, *R. norvegicus*) can breed year-round in urban areas, producing 5 litters of 4–8 pups each time (review [230]). Where rodents are prolific, some cats are likely to predate them; however, rats are large and aggressive and take skill to dispatch [231]. Two studies on the interactions between rats and cats cohabiting in alleyways in Baltimore, USA, found that stray cats preyed on juvenile but not adult rats and would scavenge freely-available refuse despite coexistence of rats in alleys [232,233]. In one study, the observed cat population did not change over a 2-year period; and although the rat population in alleys was reduced by >50% via trap-removal, the population recovered by >100% over a single year despite cat predation [233]. No studies documenting the control of pest rodents by cat predation in urban areas could be found in our review of the literature, although there are documented cases on islands for feral cats. Furthermore, cats cannot synthesize certain compounds and enzymes required for optimal health (e.g., taurine) and must consume a meat-based diet to obtain these products and fulfill their functional protein and carbohydrate requirements [234]. These nutritional requirements maintain strong selective pressure for opportunism in feeding [235], reflected in cats’ propensity to investigate new sites and approach any stimuli that may indicate prey (such as croaking frogs, cheeping baby birds, etc.; e.g., [236]). Swapping between prey types (termed ‘prey switching’) increases their breadth of diet and nutritional intake; prey switching also allows cats to survive and breed where their main prey is seasonal (e.g., rabbit breeding season [237]). Many fed cats will similarly swap between commercial food types/flavors [238,239]. The argument that stray cats will control rodents therefore ignores biological predilection for prey variety and the reproductive capacity of rodents. In addition, cat colonies with regular food sources are likely to attract pests, including raccoons, insects, possums, other cats, as well as rodents [70]. If caretakers do not clean up food remains, then rodent/pest numbers may increase and negate any argued benefit of having increased cat populations present.

### 3.11. Summary

In summary, we identified 10 ethical and welfare challenges for the management of stray cats under a TNR program.

1. Where would cats under TNR management live? Where TNR cats live has many potential consequences for whole communities. Many TNR cats are maintained in private backyards and in public spaces (e.g., schools), but many more such spaces would be needed in urban environments to save the cats euthanized annually in Australia and overseas. Maintenance of colonies in these areas does not prevent cats from roaming across whole neighborhoods.
2. Is the TNR process itself stressful? The TNR process is stressful in the short term and possibly in the long term, though studies are lacking. The stress of TNR is overlooked in favor of the potential benefits of neutering. Placing stray cats in shelters after neutering to assess temperament and implement rehabilitation procedures may increase adoptions and decrease euthanasia of strays without re-releasing cats back onto urban streets.
3. Would TNR cats be vulnerable to injury? There is overwhelming evidence for injuries to cats in urban environments. TNR cats are just as likely as roaming pets to encounter dangers, including vehicle collisions, exposure to poisons, fighting with cats and other species, and human cruelty but less likely to receive veterinary care. Increasing or maintaining the number or density of cat colonies may increase nuisance complaints and could increase acts of cruelty to cats.
4. Are stray cats vulnerable to high parasite loads and diseases? Globally, stray cats can carry high parasite loads and diseases that compromise their health. Maintaining or establishing more cat colonies increases the likelihood of pathogen transmission. TNR cats are therefore potential vectors of these to other strays, pet cats, wildlife and humans.
5. Can parasites and diseases be treated in TNR cats? And at what cost? Parasites and some diseases are difficult to effectively treat in stray cats, even when they live in colonies. It is difficult to administer effective dosages and regularity of treatments, particularly for cryptic or aggressive cats. The usefulness of carrying out incomplete regimes of worming and vaccination is questionable and a costly exercise. Without being able to administer prophylactic health care, TNR cannot guarantee the prevention of parasites and disease in colonies.
6. Are TNR cats in poor health and body condition? TNR cats can be in poor body condition without obvious physical symptoms, which compromises their short- and long-term health and welfare. Visual assessments are not adequate for assessing stray cat health. Therefore, TNR programs need to assess health using multiple methods and will require regular funds and labor.
7. What would TNR cats eat? Provision of regular food by colony caretakers does not prevent TNR cats from scavenging anthropogenic refuse that does not always provide cats with nutrition and can compromise short- and long-term health. To prevent scavenging, caretakers need to be diligent in removing refuse from colony environments, which requires extra labor.
8. Would TNR cat management impact people in urban areas? Although literature on the frequency of the transmission of some zoonotic pathogens from stray cats to humans is limited, the potential for cats to rapidly spread diseases necessitates active risk management. Stray cats are also a significant cause of public nuisance complaints, that neutering alone will not prevent. Aggressive cats can also injure people and cause allergies. These issues could preclude approval of TNR management.
9. Would TNR cat management impact pet cats? TNR cats can compromise the health and welfare of pet cats through fighting, disease and intimidation, and prioritizing TNR cats over pet cats can lead to community conflict.
10. Would TNR cat management impact urban wildlife? TNR compromises the welfare and persistence of urban wildlife and prioritizes stray cats over wildlife. It is irresponsible to introduce or maintain colonies where threatened wildlife occurs. More local research is needed on this issue.

## 4. Prevention Is Better Than Cure—Improving Responsible Pet Ownership in Australia

There is no ‘one-size-fits-all’ solution to cat overpopulation, and we do not argue that solutions are not needed. On the contrary, more research into alternative methods of stray cat population control is required (e.g., gene drives, virological contraception, etc. [240]). However, we argue that establishing TNR colonies is not an appropriate strategy for reducing euthanasia rates in the Australian context. It encourages ‘semi-ownership’, in which cats are partially provisioned by people. However, semi-owned cats rarely receive the higher levels of care often provided to pets fully owned by a household. People who feed strays often do not consider themselves owners [93,94,241]. There are several alternative approaches to reducing stray populations that attempt to address the source of stray cats, increase cat adoptions and the value placed on cats as pets. These approaches are ethically responsible and gaining traction.

### 4.1. Legislation and Community Initiatives

Australian society values cats as companions and supports legislation to improve welfare for stray cats by reducing the numbers entering shelters and living on streets. For example, a survey of 1261 Western Australians revealed strong support from both cat owners (76%) and non-owners (93%) for cat control legislation and measures such as: compulsory neutering, registering with councils, restricting cats’ ability to roam, and stipulating a maximum number of cats per property [242]. Subsequent to this study, cat-specific legislation was introduced [191], and other state governments have adopted similar legislation. Necessary complements to cat legislation include: funding and the political will to enforce bylaws, education campaigns that promote responsible ownership of cats generally, and neuter programs that target pets in areas with large stray populations (e.g., *‘The Good Neighbour Project’* by the Cat Protection Society of New South Wales [243], and National Desexing Network [244]). The public also need to know that euthanasia is not an inevitable outcome for stray cats surrendered to Australian shelters, so shelters should promote/publish their statistics to raise awareness of their efforts and successes. Zoos Victoria and the RSPCA developed a campaign to protect both wildlife and pet cats by providing owners with advice and support to keep cats safe and happy in the home environment (*‘Safe cat, Safe Wildlife’* [245]). Using mixed media, public lectures, collaboration between 10 charities and the Victorian State government, the *‘Who’s for Cats’* campaign also promotes responsible ownership by encouraging people feeding stray cats to take ownership of them (campaign offers to trap stray cats and offers discounted neutering and microchipping [246]). The concept of responsible cat ownership was also incorporated in the Victorian primary school curriculum [246]. Increasing public awareness of the issues associated with stray cats is likely to result in higher reports/surrenders of strays to shelters and councils, however, it provides managers with opportunities to get more cats off the streets and media campaigns should attract new adopters (e.g., [247]). Such holistic community initiatives demonstrate that it is possible to address the source of stray cats, increase awareness of population management issues, and improve community attitudes towards cats. However, there are limited publicly available data on how successful these community initiatives are in reducing local stray cat numbers in Australia, so this topic requires greater communication of outcomes and may require further research, either by modelling or empirical studies in specific communities.

### 4.2. Increasing Adoption

Claims for substantial population reductions in stray cats following the implementation of TNR have often included a high number of adoptions (Table 1), with some studies approaching or exceeding the 50% annual removal rates needed to reduce populations of cats by lethal control [73]. This compares to ≥75% of the TNR breeding population that needs to be neutered at all times to reduce the population [73]. Adoption can therefore be as effective as lethal control in reducing local populations and does not return cats to the environment. While removal of cats for adoption may create ‘vacuum space’ for other cats to occupy, the same is true for TNR, whether the aim is eventual population reduction or extinction.

Increasing the numbers of cats that are adopted will reduce euthanasia rates of healthy, sociable cats without incurring subsequent costs of colony feeding and maintenance. Where complaints about stray cats are made, we support investing the costs of proposed colony maintenance into trapping, housing, rehabilitating and rehoming as many stray cats as possible, with euthanasia an option only for cats with untreatable medical issues or for temperaments deemed unsuitable for rehabilitation or rehoming (10% of all admissions to Queensland RSPCA shelters in 2016 were euthanized for medical reasons, 1% euthanized for behavior [41]). While such an approach would be expensive on a large scale, so too is TNR, which still leaves cats in the environment contributing to the problems active cat management seeks to solve.

Cat ownership is relatively stable in Australia with approximately 3.3 million cats/2.3 million households in 2013 (i.e., 1.43 cats/household [248]) compared with 3.9 million cats/2.7 million households in 2016 (i.e., 1.44 cats/household [249]). Pet cats in permanent homes live extended lives, and if adoption of stray cats relies on natural attrition in the pet population, then oversupply is inevitable. More than half of Australians would like to own a pet/another pet [249]; however, barriers to ownership include an unsuitable home or lifestyle for a pet, not wanting the responsibility of ownership, pet cost, resistance by other members of a household and pet-prohibition by strata and landlords. Removing barriers to adoption for some cohorts (e.g., rental restrictions) could increase opportunities for adoption.

Methods of engaging with non-cat owners to promote adoption and prevent euthanasia have already shown progress. For example, in the past decade, RSPCA shelters have decreased the percentage of cats euthanized annually from 62% (of *n* = 69,034) to 27% (of *n* = 53,923) and increased annual rehoming from 28.5% to 57% [250]. These stellar results, and those of other shelters, are a consequence of public education, surrender counselling and adoption-drives in the traditional shelter setting (e.g., RSPCA Queensland and the Cat Haven in Western Australia trailed novel low-cost and free adult cat adoptions respectively, and found adoption-drives offering discounted adoptions successfully reduced euthanasia and increased adoptions with no adverse outcomes for cheap/free cats [251,252]. Recently, Queensland RSPCA shelters reported that the number of stray cats admitted were similar in 2011 and 2016 (total *n* = 4295 vs. 4144 [41]). However, euthanasia attributable to *“age/space limitations”* (p.11) decreased dramatically (30% vs. 2% of admissions) as a direct result of investing in improved foster systems, placing cats for adoption in pet stores and facilities of other charities. Euthanasia of ‘feral cats’ (surrendered strays and pets) also decreased when the holding-period for temperament assessment was extended from 24 to 72 h (36% vs. 22% of admissions in 2011 and 2016 respectively), giving cats more time to adjust somewhat to captivity. If these strategies and results can be reproduced in shelters across Australia, then more stray cats, including fearful individuals, can be placed in permanent homes.

### 4.3. Limiting Unplanned Breeding

Stray cats often dominate shelter admissions in Australia (e.g., [35,40]), so strategies to prevent addition to the stray population are needed, as well as neutering of the stray cats already in existence. Decreasing the numbers of pet cats that are abandoned or that breed and become stray is crucial to reducing numbers of stray cats. The most obvious method for preventing addition to stray populations from pet cats is by maintaining high neutering rates (for both sexes). Traditionally, neutering is performed from 6 months [253]; however, many cat owners do not know that females can breed from 4 months of age and do not recognize puberty/sexual behavior (e.g., lordosis in females [254,255]). Closing the two-month gap between puberty and the age of traditional neutering may help reduce the number of accidental litters born [36,256]. Pre-pubertal neutering (<4 months) is routine in shelters in Australia and overseas [257] and has been extensively studied, reviewed, and found to be safer than performing surgery on older cats (with obvious precautions taken against issues such as hypoglycemia and hyperthermia [258]) and has no adverse effects on cat growth and behavior [259]. Increasing uptake of pre-pubertal neutering amongst private veterinary practices (vs. shelters) and cat owners, new or old, may therefore prove a useful strategy for preventing the oversupply of cats in Australia. While localized neutering rates are generally high for pet cats in Australia, convenience samples suggest that neuter rates may be <50% in cats under 2 years of age [260]. There is thus plenty of scope to reduce accidental breeding by pet cats.

### 4.4. Future Investment

Some shelters and government councils euthanize cats because of a lack of resources. We argue that the money needed to implement responsible TNR programs in Australia would be better invested in holistic cat management. The facilities and services of shelters and municipal councils could be improved and increased where cat numbers are particularly high. Greater collaboration and data sharing between shelters, councils and the scientific community is needed to develop long-term community programs that actively promote responsible cat ownership and encourage adoption. Intensive trapping of stray cats in areas that pose public health threat or are of conservation concern should also be funded and not ignored for fear of reprisal by vocal groups of cat activists [7,72]. Euthanasia is an inevitable component of population control for many species. However, we believe that in Australia, stray cat euthanasia rates can be further reduced if investment is made in strategies that have already demonstrated promise. There is a clear need for economic research on the relative costs and effectiveness of different proposed strategies for reducing numbers of stray cats in Australian cities.

## 5. Conclusions

Limited *in situ* research on TNR in Australia does not justify further trials, given the myriad of potential impacts and consequences of maintaining large numbers of TNR colonies in urban areas. There is ample evidence from overseas studies that TNR programs neuter only a proportion of cats and guarantees neither colony extinction or cat health, survival or on-going care. Glaringly, substantial and constant investment is required to operate ‘responsible’ TNR programs that address the 10 ethical and welfare challenges identified by this review. With so much progress already made in Australia by stakeholders managing cats, and considering evidence for poor welfare of stray cats, threats to public health and biodiversity, as well as strong community support for cat management, we argue against introducing TNR in Australia.

We endorse the conclusions of Barrows (p.1368 [56]) for the USA, which we believe are an excellent fit to Australia’s needs: *“Ultimately, a combination of a vigorous trap and removal program; stronger and more effective licensing, identification, and confinement laws (including improved enforcement); and a massive, ongoing public education program that promotes responsible pet ownership and the necessity of keeping cats properly confined will go a long way toward reducing the number of free-roaming cats in our country.”*

On a final point, a recent attempt to introduce legal TNR in New South Wales was fully reviewed in a proposal that went to parliament in 2014 [44]; this report concluded that there was no strong evidence that TNR is successful in meeting its aims of successful stray cat management and the proposal was therefore rejected. In Australia, at least, the rejection of TNR as a management strategy for urban stray cats accounted for public sentiment in considering which management strategies are practical and ethical.

## Figures and Tables

**Table 1:** Summary of 11 published studies that present empirical data on stray Trap-Neuter-Return (TNR) colonies across the globe. These studies provide data on numbers of cats in colonies before and after the implementation of TNR programs. Numerical responses account for cat adoptions, euthanasia, disappearances, and new cats joining colonies. Data are summarized across all colonies per study; in bold are the initial and final census numbers. Numerical response cells are highlighted according to a red–green spectrum (representing high to low percentages). Percentages are rounded to the nearest whole number.

	Location	University of NSW, Australia	Chicago, USA	12 Counties, Florida, USA	Rome, Italy	University of Central Florida Campus, USA	Brooklyn, New York, USA	Park Marina, Florida, USA	Hospital, Carville, Louisiana, USA	Regent’s Park, London, UK	Hospital, Cheshire, UK	Rural Cat Colonies, Quebec, Canada
	Reference	1	2	3	4	5	6	7	8	9	10	11
Study Length (y)		9	4–10	1	2–6	11	2	1	3	1	2	1
Colony number		1	20	132	103	11	2	2	1	2	1	10
Number of individual cats (*n*)	**Initial census †**	**69**	**75**	**920**	**1655**	**155**	**129**	**80**	**41**	**17**	**76 ^A^**	**143 ^B^, median 13.5/colony**
	Neutered	55	180	643	1424	155	185	-	38	13	42	132 ^B^
	Adopted	33	59	238	-	73	5	-	0	5 ^C^	11	-
	Euthanized	21	6	-	-	17	1	47	1		12	-
	Disappeared	35	67	149	-	23 ^D^	0	-	5	1 ^E^	12	-
	Dead	15	13	151	-	10	0	-	8	0	7	-
	Other	3	0	0	-	9	0	-	0	1	0	-
	Joined	53	-	601	441	-	26	86	6	2	-	-
	**Final census †**	**15**	**44**	**678**	**1293**	**23**	**155**	**124**	**36**	**12**	**35**	**–, median 13.0/colony**
Overall num. response (%)		–78	–41	–26	–22	−79	+20	+55	–12	–29	–54	No change
Excluding adoptions (%)		–71	+175	–1	-	–72	+25	-	–12	-	–46	-

- No data. † Data in brackets are ranges (where available). **^A^** Excludes data of a known pet cat living in the colony. **^B^** Study presents averages from which numbers were calculated. **^C^** Study presented single value for adoptions/euthanasia. **^D^** Study reported nine cats relocated to ‘the woods’. **^E^** One cat escaped during TNR processing. References: 1 [43]; 2 [76]; 3 [59], 4 [80]; 5 [75]; 6 [81]; 7 [82]; 8 [77]; 9 [83]; 10 [18]; 11 [79]. Note that Bissonnette et al. [79] studied rural cat colonies while the remaining studies were for urban locations.

**Table 2:** Examples of studies published over the last decade examining the prevalence of gastrointestinal parasites in stray cats across the globe. Percentage prevalence cells are highlighted according to a red–green spectrum (representing high to low percentages). Percentages are rounded to the nearest whole number.

Study Area	*n*		Roundworms (Nematoda)										Tapeworms (Cestoda)							Protozoa					Reference
		Necropsy/Scats †	Ancylostoma braziliense *	Ancylostoma tubaeformae *	*Hookworm* spp. *	*Ollulanus tricuspis*	*Physaloptera* spp.	*Strongyloides* spp. *	Toxascaris leonina *	Toxocara cati *	*Toxocara* spp. *	*Uncinaria stenocephala*	*Diplopylidium nolleri*	Dipylidium caninum *	*Mesocestoides* spp.	*Joyeuxiella* spp.	Spirometra erinacei *	*Taenia taeniaeformis*	*Taenia hydatigena*	Cystoisospora spp. *	*Cryptosporidium* spp. *	*Giardia* spp. *	*Isospora* spp.	*Sarcocycstis* spp.	
Australia, national refuges	491	S			3%				<1%	5%				<1%			3%				3%	3%	10%		[130]
Mexico, Queretaro	273	N		1%			3%		<1%	2%				29%				3%							[131]
Portugal, Lisbon	162	N		19%		31%				38%			4%	53%		15%		3%		46%				1%	[127]
Portugal, Lisbon	231	S		1%					1%	11%		3%		1%									5%		[132]
Spain, Barcelona	50	S		4%						22%											4%	6%			[133]
Spain, Canary Islands	48	N		19%						21%				65%				31%							[134]
Italy, Milan	103	S		2%					1%	26%				1%			1%			12%					[135]
Greece, Thessaloniki	215	S			12%					18%				40%				8%							[136]
Denmark, Zealand region	92	N				13%		1%	1%	85%				1%	3%			36%							[125]
Germany, Lower Saxony	837	S			1%					27%												1%	8%		[137]
Egypt, Alexandria	170	S					1%	1%	8%	8%				19%				1%							[138]
Egypt, Kafrelsheikh	113	S		4%					5%	9%				5%				22%				2%	2%	1%	[139]
Iran, Ahvaz	140	S									45%											11%	21%	17%	[140]
Iran, Ahvaz	52	N					4%		8%	29%				23%	13%	8%		10%	2%				24%		[141]
Iran, Isfahan	131	N					37%			13%			1%		8%	76%		9%							[142]
Iran, Kashan	113	N					40%			13%		2%	65%	68%	7%	85%		15%				1%	5%	8%	[124]
Qatar, Doha	568	N		15%			5%		0%	1%								74%							[126]
UAE, Dubai	240	N			9%	1%			1%		3%		37%			66%		17%	0%						[143]
India, Aizawl Mizoram	27	N		7%			44%			59%				41%				70%							[144]
Malaysia, Kuala Lumpur	241	N	31%	35%			0%	3%		8%	24%			12%				5%							[145]
Malaysia, Georgetown	102	N	23%	20%			1%	0%		14%	37%			2%				3%							[145]
Malaysia, Kuantan	100	N	39%	33%			1%	0%		17%	42%			5%				7%							[145]
Malaysia, Malacca	100	N	31%	33%			9%	0%		5%	10%			1%				16%							[145]

† Samples were either from necropsy of the animal or from analysis of scats. * Zoonotic species.

**Table 3:** Summary of potential annual costs of prophylactic treatment for various parasites and viruses for stray cats maintained under a TNR program in Australia.

	Examples	Treatment	Requires Capture	Frequency of Treatment	Annual Cost Per Cat ^A^
Ectoparasites **^B^**	Fleas, Ticks **^C^**	Body spray	Y	Every 2 months **^D^**	$30/250ml
	Fleas	Flea collar	Y	Each lasts 8 months	$40/collar
		Tablet	N *	Daily or Weekly ^E^	$184
		Chew	N *	Monthly	$150
		Spot-on liquid	Y	Monthly	$129–182
	Fleas, Lice	Spot-on liquid	Y	Monthly	$146
G.I. Parasites **^B^**	Tapeworm	Tablet	N *	Every 3 months	$6
	Roundworms, hookworms, tapeworms	Tablet	N *	Every 3 months	$21
	Roundworms, hookworms, tapeworms, lungworms	Spot-on liquid	Y	Monthly	$150
Parasite Combinations	Fleas, ear mites, hookworms, roundworms, lungworm, heartworm **^F^**	Spot-on liquid	Y	Monthly	$150
	Fleas, ear mites, mites, hookworms, roundworms, lice, heartworms **^F^**	Spot-on liquid	Y	Monthly	$150
Viruses **^G^**	3-in-1 vaccine: Feline Enteritis, Feline Viral Rhinotracheitis, Feline Calicivirus **^H^**	Injection	Y	Annually	$14
	4-in-1 vaccine: Feline Calicivirus, Feline Herpes Virus, FPV, *Chlamydophillia felis* **^H^**	Injection	Y	Annually	$16
	5-in-1 vaccine: Feline Enteritis, Feline Viral Rhinotracheitis, Feline Calicivirus, *Chlamydophillia felis,* FeLV **^H^**	Injection	Y	Annually	$21
	FIV **^I^**	Injection	Y	Annually	$18
	FeLV **^J^**	Injection	Y	Annually	$13
	Rabies Virus **^K^**	–	–	–	–

**^A^** Prices quoted are in Australian $ at time of publication and are the cost-price of popular brands. Most treatments are administered by body mass of the animal; we have priced treatment for a 4 kg cat. **^B^** Note that some ectoparasite/wormer drugs are not safe to use on pregnant females/kittens <8 weeks of age. **^C^** Only one product in Australia claims to kill ticks. **^D^** Or every 3 weeks if treating the paralysis tick (*Ixodes holocyclus*). **^E^** Give tablets once per day for 6 days if heavy infestation, otherwise once/week. **^F^** Prevents heartworm if the cat is not already infected >2 months. **^G^** Viruses are inactivated and as described on vaccination packaging/instructions. All listed vaccinations should not be administered to pregnant cats; vaccinate cats on immunosuppressive drugs with caution. Transient post-vaccine reactions and anaphylaxis is possible but rare. **^H^** Vaccination does not prevent infection or shedding but may reduce clinical symptoms. Vaccinate healthy cats ≥8 weeks with normal body temperatures. **^I^** Vaccination actively immunizes against FIV. Vaccinate healthy cats ≥8 weeks with normal body temperatures. **^J^** Vaccination actively immunizes against FeLV. Vaccinate healthy cats ≥10 weeks with normal body temperatures. **^K^** There is currently no need for rabies vaccines in Australia. * Assumes that a cat will eat tablets in food and will consume the effective drug dose.

## Glossary

TNR	Trap–Neuter–Return (TNR) includes synonymous terminology in the literature: Trap-Neuter-Release, Trap-Neuter-Vaccinate-Return, Trap-Castrate-Return, Trap-Vasectomize-Return, and Trap-Spay-Return.
Neuter	Terminology synonymous with ‘castrate, spay, sterilize, desex’, etc.
Pet cats	Also ‘owned cats’. Cats that receive food, shelter and medical care from an owner(s).
Stray cats	*Sensu* [4]. Also ‘semi-feral, street/alley cats’, also ‘feral’ in some countries including Italy, USA, UK. Cats that are born ‘on the streets’ of urban environments and cats that were owned by people at some point but have been abandoned or become separated from their owners. Stray cats move freely within urban environments and can choose their level of association with people by adjusting their temporal or spatial activity patterns. Stray cats can be dispersed broadly across urban environments and/or be locally concentrated around specific resources such as dockyards/quays, refuse tips and bush reserves (i.e., ‘colony’ cats).
Colony cats	Also ‘fed cats’. Stray cats that are deliberately provided with food at one or more locations are classified as belonging to a ‘colony’, whether they permanently or temporarily congregate around these resources.
Feral cats	In literature from the USA and UK, stray cats in urban areas are often termed ‘feral’ (*sensu* [4]). However, in Australian literature, feral cats are totally wild animals that live and breed beyond the periphery of human settlements, surviving without human interactions or resources. Animal shelters (including some in Australia) sometimes also use the term ‘feral’ to describe cats with ‘wild’ aggressive temperaments, regardless of ownership history [40]. For consistency with Australian literature, we distinguish between stray cats in urban areas and feral cats in rural environments.
Caretakers	People who deliberately feed stray cats and may provide treatment for injuries, illness or parasites. Maintain ≥1 cat colonies that may or may not be part of organized TNR programs. Caretakers are normally female with a median age of 45–58 [59,63]. Also termed ‘semi-owners’ or ‘casual owners’.
